# Stimulant-Involved Cardiovascular Disease Mortality and Life Years Lost, 2014 to 2023

**DOI:** 10.1177/29768357251342744

**Published:** 2025-05-26

**Authors:** Rebecca Arden Harris, Sameed Ahmed M. Khatana, Dana A. Glei, Judith A. Long

**Affiliations:** 1 Cardiovascular Institute, Perelman School of Medicine, University of Pennsylvania, Philadelphia, PA, USA; 2 Leonard Davis Institute of Health Economics, University of Pennsylvania, Philadelphia, PA, USA; 3 Department of Family Medicine and Community Health, Perelman School of Medicine, University of Pennsylvania, Philadelphia, PA, USA; 4 Division of Cardiovascular Medicine, University of Pennsylvania, Philadelphia, PA USA; 5 Corporal Michael J. Crescenz VA Medical Center, Philadelphia, PA, USA; 6 Center for Population and Health, Georgetown University, Washington, DC, USA; 7 Division of General Internal Medicine, Perelman School of Medicine, University of Pennsylvania, Philadelphia, PA, USA

**Keywords:** cocaine, methamphetamines, stimulants, cardiovascular disease, mortality, age-adjusted mortality rate, average annual percent change, years of life lost

## Abstract

**Background:**

Cocaine and methamphetamine, highly cardiotoxic stimulants, are associated with increased risks of hypertension, coronary artery disease, arrhythmias, cardiomyopathy, and stroke.

**Objectives:**

This study examines trends in stimulant-involved cardiovascular disease (CVD) mortality in the U.S. from 2014 to 2023, analyzing CVD subtypes, stimulant type, population characteristics, and years of life lost (YLL).

**Design:**

Trend analysis of age-adjusted mortality rates using serial cross-section mortality data from 2014 to 2023.

**Methods:**

Using National Vital Statistics System data, we analyzed age-adjusted mortality rates (AAMRs) where CVD was the underlying cause of death and stimulants were contributing factors. We used Joinpoint regression to estimate average annual percent change (AAPC) and compare trends across groups. We calculated YLL based on age at death and demographic-specific life expectancies.

**Results:**

From 2014 to 2023, stimulant-involved CVD mortality rose sharply (AAPC: 10.1%), contrasting with stable rates of overall CVD mortality (AAPC: 0.2%). Methamphetamine-involved deaths increased faster (AAPC: 13.8%) than cocaine-involved deaths (AAPC: 6.5%). Among CVD subtypes, cerebrovascular disease showed the steepest rise (AAPC: 15.9%), followed by hypertensive (12.1%) and ischemic heart diseases (7.9%). Older adults (⩾65 years) exhibited the most pronounced increase in stimulant-involved CVD mortality (AAPC: 20.2%), while non-Hispanic American Indian/Alaska Native populations experienced the highest AAPC among racial/ethnic groups (18.1%). Stimulant-involved CVD caused nearly 1 million years of YLL, predominantly among middle-aged males (687 430 YLL) and non-Hispanic White individuals (511 120 YLL). Methamphetamine involvement (580 570 YLL) exceeded that of cocaine (423 528 YLL). Within CVD types, ischemic heart disease was the leading cause (406 248 YLL).

**Conclusions:**

Stimulant-involved CVD mortality has surged, especially among non-Hispanic American Indian/Alaska Native and non-Hispanic White populations and older adults, with cerebrovascular disease showing the largest increase among CVD subtypes. The findings reveal the importance of targeted prevention, screening, and intervention.

## Introduction

Illicit stimulants—cocaine and methamphetamines—are highly cardiotoxic, increasing oxygen demand through sympathetic activation while reducing supply via vasoconstriction, leading to ischemia, infarction, and arrhythmias.^1,2^ Even in the absence of traditional metabolic risk factors, stimulants accelerate the progression of cardiovascular disease (CVD) through myocardial remodeling, which predisposes individuals to arrhythmias and contributes to cardiomyopathy and heart failure.^3-6^ Over time, use of these agents increase the risk of hypertension, coronary artery disease, coronary vasospasm, and stroke.^7,8^ Despite the well-documented risks, the 2023 National Survey on Drug Use and Health found that cocaine and methamphetamine use remains widespread in the U.S., with 5.0 million and 2.6 million people aged 12 or older reporting past-year use, respectively.^9^ The present study describes nationwide trends in mortality rates where CVD is the underlying cause of death and cocaine or methamphetamine use is a contributing cause of death. To help characterize the role of stimulants in CVD mortality, we examine the trends by race, ethnicity, sex, age, and drug type, as well as by CVD subcategories of arrhythmia, cerebrovascular, heart failure, hypertensive, and ischemic heart diseases. To quantify the public health burden of these premature deaths, we also calculate years of life lost (YLL) within each of these categories.

## Methods

We analyzed age-adjusted mortality rates (AAMRs) using National Vital Statistics System (NVSS) 2014 to 2022 final death certificate data and 2023 provisional data.^10^ Our inclusion criteria were deaths with cardiovascular disease as the underlying cause (ICD-10: I00-I99) and cocaine (F14, R78.2, T40.5) or methamphetamine (F15, T43.6) listed as contributing causes on U.S. death certificates from 2014 to 2023. The dataset comprised all stimulant-involved cardiovascular deaths across the entire U.S. population during the study period, rather than a statistical sample, eliminating the need for sample size calculation or power analysis.

A recent study combined cases in which CVD was either a contributing or underlying cause,^11^ which prevented differentiating deaths caused by CVD from deaths primarily due to drug overdose, such as acute stimulant poisoning or opioid-related respiratory depression. To address this limitation, we focused specifically on CVD as the underlying cause, allowing for a clearer understanding of the direct impact of stimulant use on cardiovascular mortality. (See Supplemental Material for a typology of the role of stimulants in CVD mortality.) Accurate classification relies on high-quality medical documentation. CVD is a well-defined cause of death, and toxicology testing for cocaine and methamphetamine is commonly included in post-mortem screenings. However, comprehensive death investigations, including forensic autopsies, are typically conducted only when the cause of death is unclear.^12^ Incomplete medical histories, inadequate training in death certification, and reporting biases can also affect the classification of stimulant-related CVD deaths.^13,14^

We analyzed AAMR trends in stimulant-involved cardiovascular mortality by drug type (cocaine, methamphetamines), CVD subcategory (hypertensive heart diseases [ICD-10: I10-I15], ischemic heart diseases [I20-I25], and cerebrovascular diseases [I60-I69]), and demographic group (race/ethnicity, age, and sex). We used the National Cancer Institute’s (NCI) Joinpoint software (v5.2.0) to compute the average annual percent change (AAPC) for each variable, which summarized the direction and magnitude of trends over the 2014 to 2023 period. We excluded 2 CVD subcategories—stimulant-related arrhythmia and heart failure, both previously linked to stimulant use^1-6^—due to very low baseline AAMRs (<0.01 deaths per 100 000 population), which would yield unreliable AAPCs.

Pairwise comparisons of trends within categories of cardiovascular diseases, stimulant drugs, racial/ethnic groups, sex, and age were conducted using the joinpoint program’s test for parallelism. This test compares slopes and change points while controlling for different initial values and over-fitting probability. We focused pairwise comparisons on broad categories: cardiovascular disease subtypes, stimulant drugs, sex, and age groups. We omitted certain ethnic group comparisons due to fundamental differences in population size and geographic distribution (eg, non-Hispanic Asian or Pacific Islander with non-Hispanic White or Black populations). Statistical significance was set at *P* < .05 (2-tailed).

We calculated years of life lost as the difference between life expectancy and age at death.^15^ Life expectancy data were obtained from the 2019 National Center for Health Statistics (NCHS) life tables, which are stratified by age, sex, race, and Hispanic origin.^16^ These tables estimate the average reduction in life expectancy for each age group based on age at death, sex, and race/ethnicity. For each racial/ethnic group, we calculated separate male and female YLL and then summed the 2 groups and reported the total. We chose 2019 as the reference year as it marked the midpoint between 2014 and 2023 and preceded the COVID-19 pandemic’s impact on life expectancy in 2020, which moderated in 2021 and improved in 2022 and 2023.^17,18^

## Ethical Statement

This study used publicly available, de-identified data and did not require human subjects review per University of Pennsylvania IRB guidelines.

## Results

### Average Annual Percent Change

**Figure 1.**
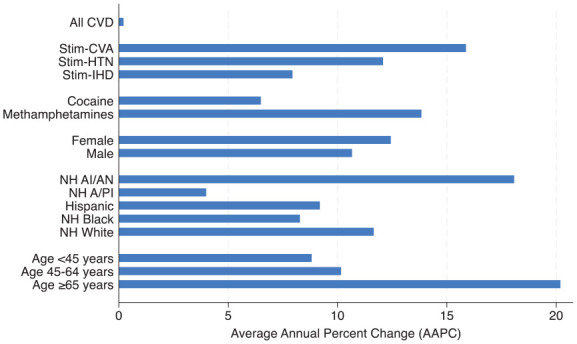
Average annual percent change (AAPC) by underlying cause of death, drug type, and demographic group, 2014 to 2023. CVD was the underlying cause of death for all categories. For All CVD, this includes deaths with and without stimulants as a contributing cause. For all other categories, stimulants were a contributing cause of death. Abbreviations: AAPC, average annual percent change; NH A/PI, non-Hispanic Asian or Pacific Islander; NH AI/AN, non-Hispanic American Indian or Alaska Native; CVD, cardiovascular disease; NH Black, non-Hispanic Black; NH White, non-Hispanic White; Stim-CVA, stimulant-involved cerebrovascular disease; Stim-HTN, stimulant-involved hypertension disease; Stim-IHD, stimulant-involved ischemic heart disease.

Table 1 reports the average annual percent change (AAPC) in age-adjusted mortality rates from 2014 to 2023. While overall CVD mortality rates remained stable over this period (AAPC 0.2%), there was a steep increase in CVD deaths where a stimulant was a contributing cause (AAPC 10.1%). Among stimulant-involved deaths, the AAPC increase was highest for cerebrovascular diseases (15.9%), followed by hypertensive (12.1%), and ischemic heart diseases (7.9%). The AAPC for cocaine involvement (6.5%) was markedly lower than for methamphetamine involvement (13.8%; Figure 1).

**Table 1.** Age-adjusted cardiovascular disease (CVD) mortality rates, average annual percent change (AAPC), and years of life lost (YLL) by CVD subcategory, sex, race/ethnicity, stimulant drug type, and age group: 2014 to 2023.

	Age-adjusted mortality per 100 000 (AAMR)		Percent change in AAMR	Average annual percent change (AAPC)	Number of deaths	Years of life lost (YLL)
Category	2014	2023	2014-2023	2014-2023	2014-2023	2014-2023
All CVD^a^	219.88	218.34	−0.70	0.21	8 805 618	104 315 727
Stim-CVD^b^	0.56	1.32	135.71	10.10^c^	34 309	954 627
Stim-CVA	0.06	0.20	233.33	15.87^c^	4824	145 822
Stim-HTN	0.11	0.28	154.55	12.08^c^	7079	200 961
Stim-IHD	0.28	0.56	100.00	7.94^c^	15 002	406 248
Drug type^b,d^						
Cocaine	0.30	0.54	80.00	6.49^c^	15 352	423 528
Methamphetamines	0.25	0.83	232.00	13.83^c^	19 625	580 570
Sex^b^						
Female	0.25	0.69	176.00	12.43^c^	8426	267 197
Male	0.86	2.01	133.72	10.66^c^	25 883	687 430
Race/ethnicity^b^						
NH AI/AN	1.15	4.51	292.17	18.07^c^	695	27 282
NH A/PI^e^	0.32	0.40	25.00	3.99^c^	768	19 298
Hispanic	0.48	1.14	137.50	9.19^c^	4292	139 835
NH Black	1.42	2.76	94.37	8.28^c^	9604	227 976
NH White	0.44	1.18	168.18	11.65^c^	18 360	511 120
Age (years)^b^						
<45	0.24	0.46	91.67	8.81^c^	6566	280 726
45-64	1.66	3.75	125.90	10.16^c^	23 028	626 933
⩾65	0.30	1.46	386.67	20.19^c^	4711	76 450

Abbreviations: AAMR, age-adjusted mortality rate; AAPC, average annual percent change; NH A/PI, non-Hispanic Asian or Pacific Islander; NH AI/AN, non-Hispanic American Indian or Alaska Native; CVD, cardiovascular disease; NH Black, non-Hispanic Black; NH White, non-Hispanic White; Stim-CVA, stimulant-involved cerebrovascular disease; Stim-CVD, stimulant-involved cardiovascular disease; Stim-HTN, stimulant-involved hypertension disease; Stim-IHD, stimulant-involved ischemic heart disease.

a Includes all deaths where CVD was the underlying cause, whether or not stimulants were a contributing cause.

b Rates refer to stimulant-involved deaths.

c *P* ⩽ .05.

d There is an overlap in the number of deaths and years of life lost (YLL) between cocaine and methamphetamines, as both drugs may be listed on a decedent’s death certificate. Approximately 1.95% of the total stimulant-involved CVD deaths involve both substances.

e NVSS reports combined non-Hispanic Asian/Pacific Islander (A/PI) data in the initial years (2014-2017) of the study period; however, the 2019 CDC life expectancy tables report estimates only for non-Hispanic Asians, excluding Pacific Islanders. Since non-Hispanic Asians make up over 95% of the NH A/PI group, the resulting inaccuracy is expected to be minimal (Arias and Xu^16^).

The AAPC was highest for those age 65 years and older (20.2%) than for those under 45 (8.8%) and for those aged 45 to 64 (10.2%). The AAPC for males (10.7%) was slightly lower than for females (12.4%). Among racial/ethnic groups, non-Hispanic American Indian/Alaska Native populations had the highest AAPC (18.1%), followed by non-Hispanic White (11.6%), Hispanic (9.2%), non-Hispanic Black (8.3%), and non-Hispanic Asian/Pacific Islander populations (4.0%). Notably, non-Hispanic Black individuals experienced the highest *absolute* death rates at the outset of the study period (1.4 per 100 000 in 2014), and non-Hispanic American Indian/Alaska Native (4.5) and non-Hispanic Black individuals (2.7) had the highest mortality rates at the end of the study period in 2023 (Table 1).

### Pairwise Comparison of AAPCs

Table 2 presents the pairwise comparisons of AAPCs. Significant differences were found between overall cardiovascular disease mortality trends and stimulant-related CVD mortality trends (*P* < .001), and among stimulant-related subcategories: hypertensive heart diseases versus ischemic heart diseases (*P* = .022), hypertensive heart diseases versus cerebrovascular diseases (*P* = .004), and cerebrovascular versus ischemic heart diseases (*P* = .037). AAPC differences in mortality trends were also significant between cocaine and methamphetamine involvement (*P* = .002). Among racial/ethnic groups, there were differences between Hispanic and non-Hispanic White individuals (*P* = .011), non-Hispanic White and non-Hispanic Black individuals (*P* = .001), and non-Hispanic American Indian/Alaska Native and non-Hispanic Asian/Pacific Islander individuals (*P* = .014). However, the difference between Hispanic and non-Hispanic Black individuals was not significant (*P* = .789). Age comparisons revealed differences in AAPCs between those aged 45 to 64 years and those aged 65 years and older (*P* < .001), as well as between those under 45 years and those aged 65 years and older (*P* = .002). The comparison between those under 45 years and those aged 45 to 64 years was non-significant (*P* = .574). There was no significant difference in AAPCs between females and males (*P* = .371).

**Table 2.** Pairwise comparisons of average annual percent changes (AAPCs) using test of parallelism between groups.

Pairwise comparisons	*P*-value
All CVD vs Stim-CVD	.0002*
Stim-HTN vs Stim-IHD	.0218*
Stim-HTN vs Stim-CVA	.0038*
Stim-CVA vs Stim-IHD	.0371*
Cocaine vs Methamphetamines	.0022*
Female vs Male	.3709
NH White vs Hispanic	.0107*
Hispanic vs NH Black	.7893
NH White vs NH Black	.0013*
NH AI/AN vs NH A/PI	.0136*
<45 years vs 45-64 years	.5736
45-64 y vs ⩾ 65 y	.0002*
<45 y vs ⩾ 65 y	.0018*

Abbreviations: AAPC, average annual percent change; NH A/PI, non-Hispanic Asian or Pacific Islander; NH AI/AN, non-Hispanic American Indian or Alaska Native; CVD, cardiovascular disease; NH Black, non-Hispanic Black; NH White, non-Hispanic White; Stim-CVA, stimulant-involved cerebrovascular disease; Stim-CVD, stimulant-involved cardiovascular disease; Stim-HTN, stimulant-involved hypertension disease; Stim-IHD, stimulant-involved ischemic heart disease.

* Statistically significant at *P* ⩽ .05.

### Years of Lost Life

**Figure 2.**
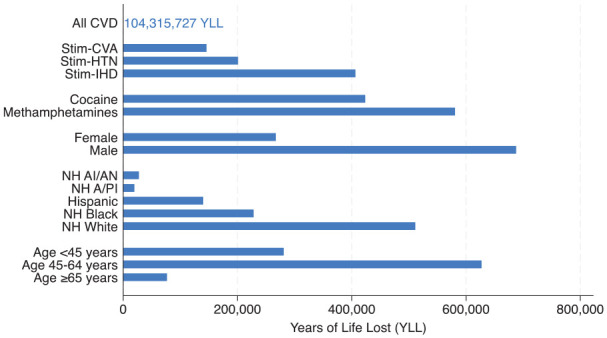
Years of life lost (YLL) by underlying cause of death, drug type, and demographic group, 2014 to 2023. CVD was the underlying cause of death for all categories. For All CVD, this includes deaths with and without stimulants as contributing causes. For all other categories, stimulants were a contributing cause of death. YLL for All CVD (104 315 727) is shown numerically rather than as a bar to avoid expanding the YLL axis scale to 104 million, which would obscure the differences among the stimulant-specific and demographic categories. Abbreviations: AAPC, average annual percent change; NH A/PI, non-Hispanic Asian or Pacific Islander; NH AI/AN, non-Hispanic American Indian or Alaska Native; CVD, cardiovascular disease; NH Black, non-Hispanic Black; NH White, non-Hispanic White; Stim-CVA, stimulant-involved cerebrovascular disease; Stim-HTN, stimulant-involved hypertension disease; Stim-IHD, stimulant-involved ischemic heart disease.

Between 2014 and 2023, cardiovascular disease as the underlying cause of death resulted in a total of 104 315 727 YLL, with stimulant-involved CVD accounting for 954 627 YLL, or 0.90% (Table 1). Males experienced greater losses from stimulant-involved CVD, totaling 687 430 YLL, compared to 267 197 YLL among females. Non-Hispanic White individuals had the highest YLL at 511 120, followed by non-Hispanic Black individuals with 227 976 YLL. Hispanic populations lost 139 835 years, while non-Hispanic American Indian/Alaska Native and non-Hispanic Asian or Pacific Islander populations lost 27 282 years and 19 298 years, respectively. Among age groups, individuals aged 45 to 64 experienced the most losses, totaling 626 933 YLL. Those under 45 lost 280 726 years, while individuals aged 65 and older lost 76 450 years (Figure 2).

By CVD subtype, ischemic heart disease was responsible for 406 248 YLL, hypertensive disease for 200 961 YLL, and cerebrovascular disease for 145 822 YLL. Methamphetamines were associated with 580 570 YLL, while cocaine was a contributing cause in 423 528 YLL. Some deaths involved both substances, resulting in double counting of both deaths and YLL. Approximately 1.95% of stimulant-involved CVD deaths listed both methamphetamines and cocaine on the death certificate.

## Discussion

This study identified a troubling divergence in cardiovascular disease mortality trends. While overall CVD mortality rates have remained stable over the past decade, CVD deaths involving stimulants have surged, especially among non-Hispanic American Indian/Alaska Native, non-Hispanic White, and older adults. The analysis was guided by our typology (Supplemental Material) that distinguished CVD mortality cases involving stimulants from drug poisoning deaths. The typology directed our focus toward cases where CVD was the underlying cause of death, thereby defining the specific set of cases for the trend analysis, pairwise comparisons of AAPCs across demographic groups, CVD subcategories, and stimulant types, as well as the assessment of years of life lost.

The pairwise comparisons displayed significant differences in stimulant-related CVD mortality. Methamphetamine use showed a steeper increase in mortality rates compared to cocaine, while stimulant-related ischemic heart disease, hypertension, and cerebrovascular disease exhibited distinct trends. These findings expose gaps in our understanding and suggest promising areas for future research.

Among the demographic groups examined, age-related patterns warrant particular attention. The seemingly paradoxical finding of a higher AAPC but lower YLL in adults aged 65 and older stems from 2 factors. First, lower baseline mortality rates mean that even small absolute increases in deaths produce large percentage changes. Second, shorter remaining life expectancy inherently limits potential YLL. Similarly, high AAPCs in certain groups, such as the Non-Hispanic American Indian/Alaska Native population, can coincide with relatively low YLL due to smaller population sizes. These patterns illustrate the importance of interpreting AAPC alongside absolute mortality burden, taking into account differences in population size and life expectancy, as percentage increases alone can be misleading.

The YLL analysis revealed the profound impact of stimulant-related cardiovascular mortality. Between 2014 and 2023, nearly 1 million years of life were lost. Middle-aged individuals, males, and non-Hispanic White populations bore the greatest absolute losses, with methamphetamine use and ischemic heart disease as the leading drivers. At current rates, by 2030 we can expect an additional 835 000 YLL (30 000 deaths) from stimulant-involved CVD. These projections represent only a fraction of the total impact; individuals who continue to use stimulants remain at elevated risk for future mortality and a range of other health complications, including infections, metabolic disorders, neurological conditions, psychiatric illnesses, and renal dysfunction.^3^

### Limitations

This study is limited by potential miscoding of death certificates and incomplete documentation of drug-related details.^19-21^ In addition, testing protocols vary by jurisdiction, with some lacking comprehensive diagnostic or toxicology screenings for suspected cardiovascular deaths due to resource limitations or policy differences. Moreover, post-mortem detection windows for stimulants in blood are brief using standard toxicology panels, making delayed or unavailable testing a potential source of underestimation.^22,23^ Stigma surrounding drug use may further discourage accurate reporting,^24^ while incomplete medical histories, particularly in the absence of documented drug use, can obscure the role of stimulants in these deaths.^25^ Additionally, some stimulant-related deaths may result from long-term cardiovascular damage rather than recent use, further complicating identification. Together, these factors could contribute to an underestimation of stimulant-related CVD mortality.

Another study limitation concerns the estimation of YLL. The YLL metric represents an upper bound of the impact of stimulant-related CVD mortality. This is because decedents in each demographic group probably had a higher prevalence of comorbidities or other risk factors that contributed to increased mortality compared to the general population. These additional health conditions could independently lower life expectancy, potentially overstating YLL specifically attributable to stimulant-related CVD.

## Conclusions

By identifying patterns across drug types and cardiovascular conditions, this study clarifies how stimulant use contributes to cardiovascular mortality. The exponential rise in stimulant-involved CVD deaths—particularly among American Indian/Alaska Native populations, older adults, and methamphetamine users—calls for urgent action. Although cardiovascular disease remains the leading cause of death in the United States,^17^ overall CVD mortality has remained stable over the past decade (0.2% AAPC). The disproportionate impact on middle-aged adults (626 933 YLL) and the diverging trends between cocaine (6.5% AAPC) and methamphetamine (13.8% AAPC) emphasize the need for tailored interventions at both clinical and policy levels.

Reversing this trend requires increased funding for culturally relevant prevention programs and expanded clinical interventions. Cardiologists should screen for stimulant use, particularly in high-risk groups and patients with cerebrovascular (15.9% AAPC), hypertensive (12.1%), and ischemic heart diseases (7.9%). Addiction medicine specialists must improve access to evidence-based treatments, including contingency management, cognitive-behavioral therapy, and emerging pharmacological options.^26-28^ Primary care clinicians should lead prevention through routine screening and early intervention, especially for older adults, whose mortality rates have risen sharply (20.2% AAPC for those 65 and older).

The human toll of these preventable deaths—the lost opportunities for families, the absence of loved ones, and the lasting void in their communities—reinforces the urgency of a coordinated response. By prioritizing the populations bearing the greatest burden, we can build sustainable systems to prevent and address stimulant-involved cardiovascular disease across all communities.

## Supplemental Material

sj-docx-1-sat-10.1177_29768357251342744 – Supplemental material for Stimulant-Involved Cardiovascular Disease Mortality and Life Years Lost, 2014 to 2023 Supplemental material, sj-docx-1-sat-10.1177_29768357251342744 for Stimulant-Involved Cardiovascular Disease Mortality and Life Years Lost, 2014 to 2023 by Rebecca Arden Harris, Sameed Ahmed M. Khatana, Dana A. Glei and Judith A. Long in Substance Use: Research and Treatment

## References

[1] Tobolski J Sawyer DB Song SJ Afari ME . Cardiovascular disease associated with methamphetamine use: a review. Heart Fail Rev. 2022;27(6):2059-2065. 35844009 10.1007/s10741-022-10261-7

[2] Phillips K Luk A Soor GS , et al. Cocaine cardiotoxicity: a review of the pathophysiology, pathology, and treatment options. Am J Cardiovasc Drugs. 2009;9(3):177-196. 19463023 10.2165/00129784-200909030-00005

[3] Clinical Guideline Committee (CGC) Members; ASAM Team; AAAP Team; IRETA Team. The ASAM/AAAP clinical practice guideline on the management of stimulant use disorder. Additional resources: evidence to decision tables, summary. J Addict Med. 2024;18(1S Suppl 1):1-56. 10.1097/ADM.0000000000001299 PMC11105801 38669101

[4] Coffin PO Suen LW . Methamphetamine toxicities and clinical management. NEJM Evid. 2023;2(12):EVIDra2300160. 10.1056/EVIDra2300160 PMC11458184 38320504

[5] Davis JD Bepo L Suen LW , et al. Implementing Heart Plus: design and early results of a novel co-management clinic for patients with stimulant-associated cardiomyopathy. J Card Fail. 2024;30(7):869-876. 37984791 10.1016/j.cardfail.2023.10.481

[6] Manja V Nrusimha A Gao Y , et al. Methamphetamine-associated heart failure: a systematic review of observational studies. Heart. 2023;109(3):168-177. 36456204 10.1136/heartjnl-2022-321610

[7] Riley ED Hsue PY Coffin PO . A chronic condition disguised as an acute event: the case for re-thinking stimulant overdose death. J Gen Intern Med. 2022;37(13):3462-3464. 35713806 10.1007/s11606-022-07692-1 PMC9550944

[8] Curran L Nah G Marcus GM , et al. Clinical correlates and outcomes of methamphetamine-associated cardiovascular diseases in hospitalized patients in California. J Am Heart Assoc. 2022;11(16):e023663. 10.1161/JAHA.121.023663 PMC9496303 35912709

[9] Substance Abuse and Mental Health Services Administration. 2022 National Survey on Drug Use and Health: Detailed Tables. Published 2023. Accessed June 1, 2024. https://www.samhsa.gov/data/report/2022-nsduh-detailed-tables

[10] Centers for Disease Control and Prevention, National Center for Health Statistics. National vital statistics system, provisional mortality on CDC WONDER Online Database. Accessed May 26, 2024. http://wonder.cdc.gov/mcd-icd10-provisional.html.

[11] Minhas AMK Kewcharoen J Hall ME , et al. Temporal trends in substance use and cardiovascular disease-related mortality in the United States. J Am Heart Assoc. 2024;13(2):e030969. 10.1161/JAHA.123.030969 PMC10926834 38197601

[12] National Center for Health Statistics. Physician’s Handbook on Medical Certification of Death. National Center for Health Statistics. 2023. doi:10.15620/cdc:131005.

[13] Hoffman RA Venugopalan J Qu L Wu H Wang MD . Improving validity of cause of death on death certificates. ACM BCB. 2018;2018:178-183. 32558825 10.1145/3233547.3233581 PMC7302107

[14] Morgan A Andrew T Guerra SMA , et al. Provider reported challenges with completing death certificates: a focus group study demonstrating potential sources of error. PLoS One. 2022;17(5):e0268566. 10.1371/journal.pone.0268566 PMC9122187 35594279

[15] Rodzlan Hasani WS Muhamad NA Hanis TM , et al. The burden of premature mortality from cardiovascular diseases: a systematic review of years of life lost. PLoS One. 2023;18(4):e0283879. 10.1371/journal.pone.0283879 PMC10121009 37083866

[16] Arias E Xu JQ . United States Life Tables, 2019. National Vital Statistics Reports; vol 70 no 19. National Center for Health Statistics; 2022. doi:10.15620/cdc:113096. 35319436

[17] Arias E Kochanek KD Xu JQ Tejada-Vera B . Provisional Life Expectancy Estimates for 2022. Vital Statistics Rapid Release; no 31. National Center for Health Statistics; November 2023. doi:10.15620/cdc:133703.

[18] Murphy SL Kochanek KD Xu J Arias E . Mortality in the United States, 2023. NCHS Data Brief. 2024;521. doi:10.15620/cdc/170564 PMC11770397 39819663

[19] Davis GG Cadwallader AB Fligner CL , et al. Position Paper: Recommendations for the investigation, diagnosis, and certification of deaths related to opioid and other drugs. Am J Forensic Med Pathol. 2020;41(3):152-159. 32404634 10.1097/PAF.0000000000000550

[20] Shearer RD Shippee ND Winkelman TNA . Characterizing trends in methamphetamine-related health care use when there is no ICD code for "methamphetamine use disorder. J Subst Abuse Treat. 2021;127:108369. 34134872 10.1016/j.jsat.2021.108369 PMC8217729

[21] Kariisa M Seth P Scholl L Wilson N Davis NL . Drug overdose deaths involving cocaine and psychostimulants with abuse potential among racial and ethnic groups – United States, 2004–2019. Drug Alcohol Depend. 2021;227:109001. 34492555 10.1016/j.drugalcdep.2021.109001

[22] Dalsasso LCF Marchioni C . Post-mortem toxicological analysis of cocaine: main biological samples and analytical methods. Forensic Sci Med Pathol. 2024;20(3):1091-1101. 37553490 10.1007/s12024-023-00678-3

[23] Lewis D Kenneally M van denHeuvel C Byard RW . Methamphetamine deaths: changing trends and diagnostic issues. Med Sci Law. 2021;61(2):130-137. 10.1177/0025802420986707 33423599

[24] Forchuk C Serrato J Scott L . Perceptions of stigma among people with lived experience of methamphetamine use within the hospital setting: qualitative point-in-time interviews and thematic analyses of experiences. Front Public Health. 2024;12:1279477. 38414902 10.3389/fpubh.2024.1279477 PMC10896942

[25] Korona-Bailey JA Nechuta S Golladay M , et al. Characteristics of fatal opioid overdoses with stimulant involvement in Tennessee: a descriptive study using 2018 State Unintentional Drug Overdose Reporting System Data. Ann Epidemiol. 2021;58:149-155. 33744415 10.1016/j.annepidem.2021.03.004

[26] Minozzi S Saulle R Amato L Traccis F Agabio R . Psychosocial interventions for stimulant use disorder. Cochrane Database Syst Rev. 2024;2(2):CD011866. 10.1002/14651858.CD011866.pub3 PMC10867898 38357958

[27] Clinical Guideline Committee (CGC) Members, ASAM Team, AAAP Team, IRETA Team. The ASAM/AAAP clinical practice guideline on the management of stimulant use disorder. J Addict Med. 2024;18(1S Suppl 1):1-56. 10.1097/ADM.0000000000001299 PMC11105801 38669101

[28] Montoya ID Volkow ND . IUPHAR review: new strategies for medications to treat substance use disorders. Pharmacol Res. 2024;200:107078. 38246477 10.1016/j.phrs.2024.107078 PMC10922847

