# Supplementary material for: Stimulant-Involved Cardiovascular Disease Mortality and Life Years Lost, 2014 to 2023
Source: Subst Use. 2025 May 26;19:29768357251342744. doi: 10.1177/29768357251342744 (PMC12106991; doi:10.1177/29768357251342744)
Supplement: sj-docx-1-sat-10.1177_29768357251342744 – Supplemental material for Stimulant-Involved Cardiovascular Disease Mortality and Life Years Lost, 2014 to 2023 [file sj-docx-1-sat-10.1177_29768357251342744.docx]

**Stimulant-Involved Cardiovascular Disease Mortality and Life Years Lost, 2014–2023**

**Supplement**

**
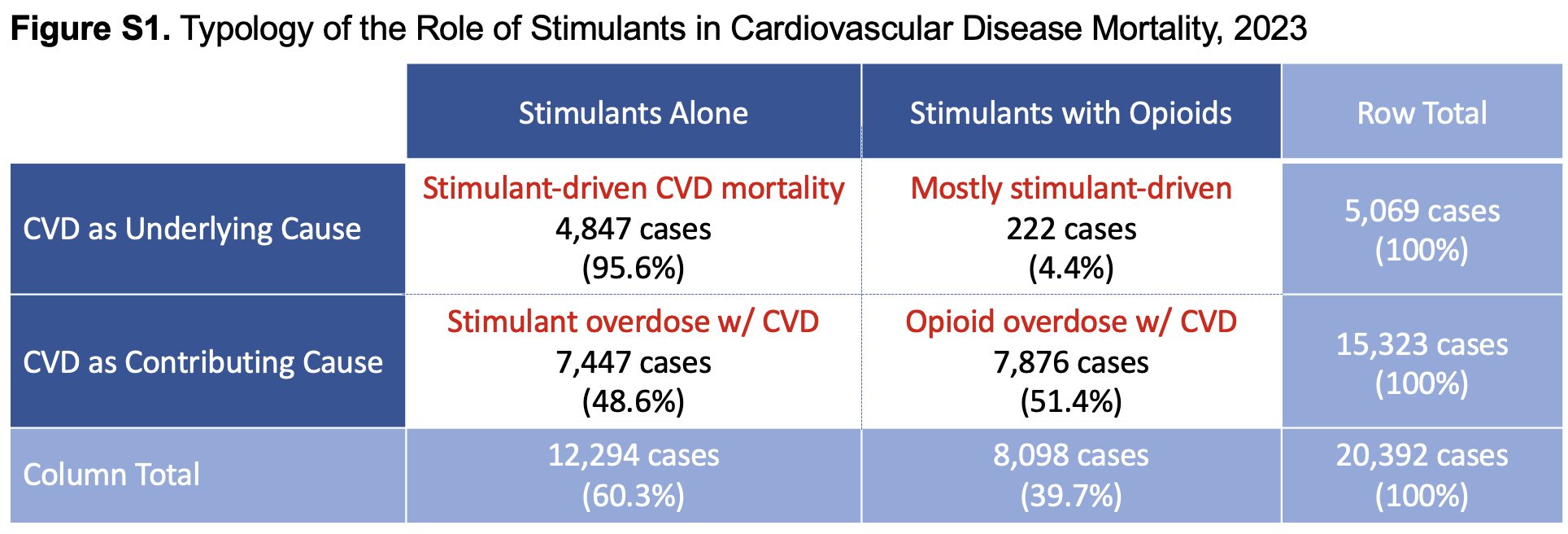
**

We developed a typology to investigate the relationship between stimulant use and cardiovascular disease (CVD) mortality using 2023 data from the National Vital Statistics System (NVSS).^1^ A typology is a classification system that organizes complex phenomena into distinct categories, typically through a cross-classification of nominal-scale variables.^2^ This method is used to create conceptual categories that show the similarities and differences among cases while providing an inventory of possible types. The 2x2 typology in Figure S1 categorizes deaths by substance type (stimulants alone vs. stimulants combined with opioids) and cause of death classification (underlying vs. contributing). This classification scheme helps identify deaths most directly attributable to the cardiotoxic effects of stimulants and avoids conflation with overdose-related deaths, which often involve multiple substances and contributing factors.

The typology reveals significant differences in category frequencies (P<0.001, calculated using the chi-square test). When CVD is the underlying cause of death, 95.6% of cases involve stimulants alone, compared to 4.4% involving both stimulants and opioids. Conversely, when CVD is a contributing factor, cases are nearly evenly split: 48.6% driven by stimulant overdose and 51.4% by opioid overdose. This shows the distinct contributions of stimulants and opioids to cardiovascular mortality, depending on whether CVD is the primary or secondary cause. We note that the cell labels "Stimulant overdose with CVD" and "Opioid overdose with CVD" are somewhat imprecise; they include deaths with non-CVD, non-overdose underlying causes such as infections, neoplasms, digestive diseases, respiratory diseases, and endocrine disorders, which collectively account for 18.6% of all deaths where CVD is listed as a contributing factor.

Stimulants, such as cocaine and methamphetamines, directly stimulate the sympathetic nervous system, leading to increased heart rate, blood pressure, myocardial oxygen demand, and vasoconstriction.^3^ These physiological effects can precipitate acute cardiovascular events, including ischemia, arrhythmias, myocardial infarction, and cerebrovascular events like stroke.^4^ In contrast, opioids primarily cause harm through respiratory depression, which leads to hypoxia.^5^ Severe oxygen deprivation can result in cascading physiological stress and cardiac compromise, such as ischemic events or arrhythmias, as a secondary consequence of respiratory failure. This distinction highlights the direct cardiotoxic mechanisms of stimulants versus the indirect pathways of cardiovascular injury associated with opioids.

In cases of acute stimulant toxicity, the cause of death may be more difficult to determine.^6^ Most deaths attributed to acute stimulant toxicity, when opioids are not involved, have no additional cause of death listed. However, a large post-mortem study of methamphetamine deaths in San Francisco, California, revealed that 20% of decedents had a cardiac cause of death, 55% had a cerebrovascular cause, and 27% had significant cardiac conditions listed as contributing factors.^7^ In cases where no additional cause was recorded, fatal cardiac arrhythmias were probably the cause of death. These arrhythmias may not be detectable and would not necessarily be suspected without supporting medical records or autopsy findings. When both CVD and stimulants are contributing causes of death, it becomes challenging to distinguish their specific roles. These cases appear to reflect a complex interplay of stimulant toxicity, cerebrovascular involvement, and pre-existing cardiovascular conditions, making it difficult to attribute a single underlying cause.

To our knowledge, this is the first typology to classify stimulant-related CVD mortality, which we used to define our study domain: deaths in which CVD was the underlying cause and stimulants were a contributing factor.^[[1]](#footnote-1)^* The typology shows the importance of distinguishing between the direct cardiotoxic effects of stimulants as an underlying cause and the multifactorial pathways contributing to mortality when CVD is a contributing cause, allowing for a clearer understanding of how stimulants directly contribute to cardiovascular mortality. This distinction is crucial for designing prevention strategies and guiding future research.

**References**

1. Centers for Disease Control and Prevention, National Center for Health Statistics. National Vital Statistics System, Provisional Mortality on CDC WONDER Online Database. Data are from the final Multiple Cause of Death Files, 2018-2022, and from provisional data for years 2023-2024, as compiled from data provided by the 57 vital statistics jurisdictions through the Vital Statistics Cooperative Program. Accessed at <http://wonder.cdc.gov/mcd-icd10-provisional.html>.

2. Bailey KD (1994). Typologies and taxonomies: An introduction to classification techniques. Sage Publications, Inc. 1994; volume 102, Series: Quantitative Applications in the Social Sciences

3. Coffin PO, Suen LW. Methamphetamine Toxicities and Clinical Management. NEJM Evid. 2023 Dec;2(12):EVIDra2300160. doi: 10.1056/EVIDra2300160. Epub 2023 Nov 28. PMID: 38320504; PMCID: PMC11458184.

4. Tsatsakis A, Docea AO, Calina D, Tsarouhas K, Zamfira LM, Mitrut R, Sharifi-Rad J, Kovatsi L, Siokas V, Dardiotis E, et al. A mechanistic and pathophysiological approach for stroke associated with drugs of abuse. J Clin Med. 2019;8:1295. doi: 10.3390/jcm8091295

5. Montandon G. The pathophysiology of opioid-induced respiratory depression. Handb Clin Neurol. 2022;188:339-355. doi: 10.1016/B978-0-323-91534-2.00003-5. PMID: 35965031.

6. Hughto JMW, Kelly PJA, Vento SA, Pletta DR, Noh M, Silcox J, Rich JD, Green TC. Characterizing and responding to stimulant overdoses: Findings from a mixed methods study of people who use cocaine and other stimulants in New England. Drug Alcohol Depend. 2024 Nov 19;266:112501. doi: 10.1016/j.drugalcdep.2024.112501. Epub ahead of print. PMID: 39608288.

7. Turner C, Chandrakumar D, Rowe C, Santos GM, Riley ED, Coffin PO. Cross-sectional cause of death comparisons for stimulant and opioid mortality in San Francisco, 2005–2015. Drug Alcohol Depend 2018;185:305-312. DOI: 10.1016/j.drugalcdep.2017.12.030.

8. Kelly BC, Vuolo M. Trends in Psychotropic Drug-Implicated Cardiovascular Mortality: Patterns in U.S. Mortality, 1999-2020. Am J Prev Med. 2023 Sep;65(3):377-384.

1. * An earlier study with similar case selection criteria excluded key ICD codes—F14 for cocaine use disorder and F15 for other stimulant use disorders, predominantly methamphetamine—and consequently captured only 45% of cases where CVD was the underlying cause and stimulants were a contributing factor.^8^ [↑](#footnote-ref-1)
